# Supplementary material for: Mouse spermatozoa with higher fertilization rates have thinner nuclei
Source: PeerJ. 2017 Oct 12;5:e3913. doi: 10.7717/peerj.3913 (PMC5641427; doi:10.7717/peerj.3913)
Supplement: Supplemental Information 1 — Figure S1. Conventional method of sperm head shape quantification Parameters such as length, width, and angle are usually obtained by intuitively selecting points and measuring them. But these parameters have a risk to underestimate the complex shape of sperm head contour. Figure S2. Acrosome reactions did not alter sperm nucleus morphology. Images show the Hoechst-stained sperm nucleus (A), the acrosome-reacted Acr-EGFP (B), and the two images merged (C) following the spontaneous occurrence of an acrosome reaction after a 7-h incubation in TYH medium. In spermatozoa from Acr-EGFP mice (Nakanishi et al., 2004), the presence (left sperm in B and C) or absence (right sperm in B and C) of EGFP expression indicates intact or reacted acrosomes, respectively. (D) The aspect ratios of acrosome-intact (n = 44) and -reacted sperm (n = 46) were not significantly different (unpaired, two-tailed t-test; P = 0.12). The error bars indicate the standard deviations from the mean. Figure S3. Hoechst-stained nuclei accurately represented the sperm head contour. Representative images show the head morphology (left panels) of two spermatozoa with Hoechst-stained nuclei (right panels). The contours of the sperm heads overlap completely with the Hoechst-stained nuclei. Figure S4. Elliptic Fourier descriptors accurately approximated the sperm head shape. (A) N denotes the number of ellipses that were used to approximate the sperm head contour (black line; see also Eq. (1)). The approximated contour (red line) became more accurate as the N increased. (B) An enlarged view at N = 20 shows that the overlap of red and black lines indicating a precise approximation. Figure S5. The inclusion of abnormal spermatozoa affected the principal component score. (A) Principle component analysis (PCA) was performed of B6N cauda epididymis-isolated spermatozoa without exclusion of abnormal spermatozoa (n = 179). The standard deviation (SD) of the sperm head contours in the first principal component (PC1) [file peerj-05-3913-s001.pdf]

S1 Figure

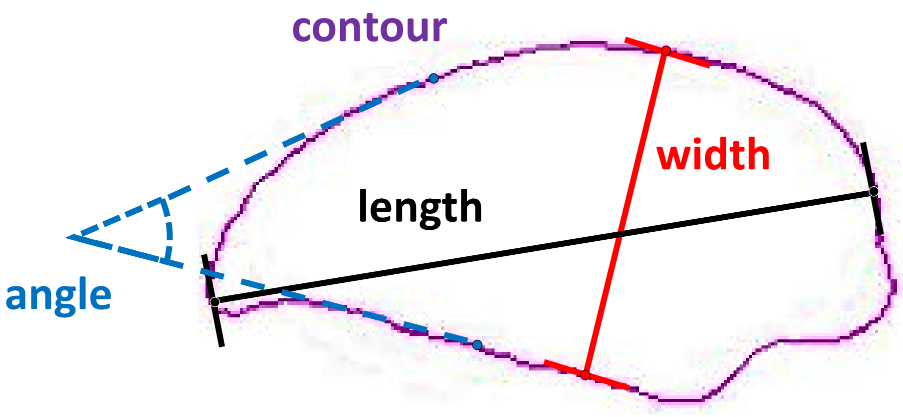

S2 Figure

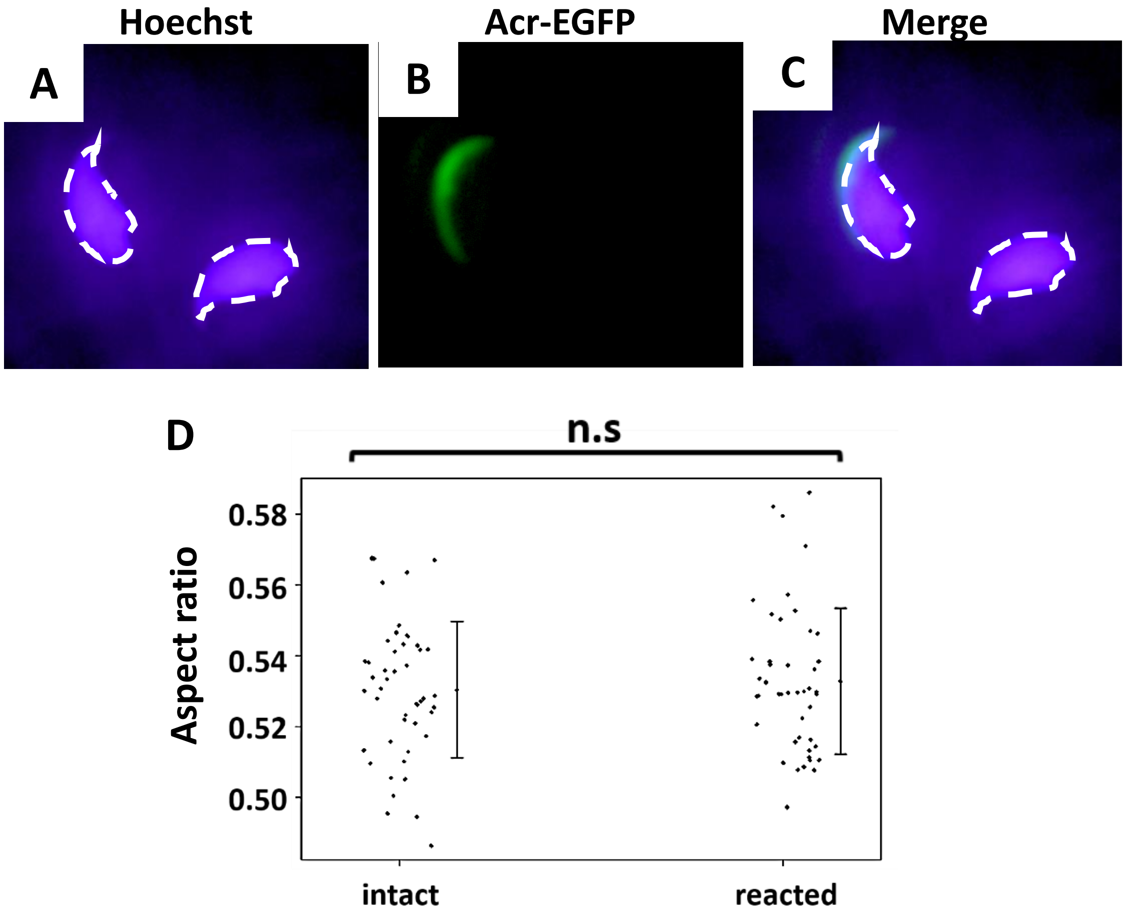
Hoechst
Acr-EGFP
Merge
A
B
C
D
n.s
Aspect ratio
0.58
0.56
0.54
0.52
0.50
intact
reacted

S3 Figure

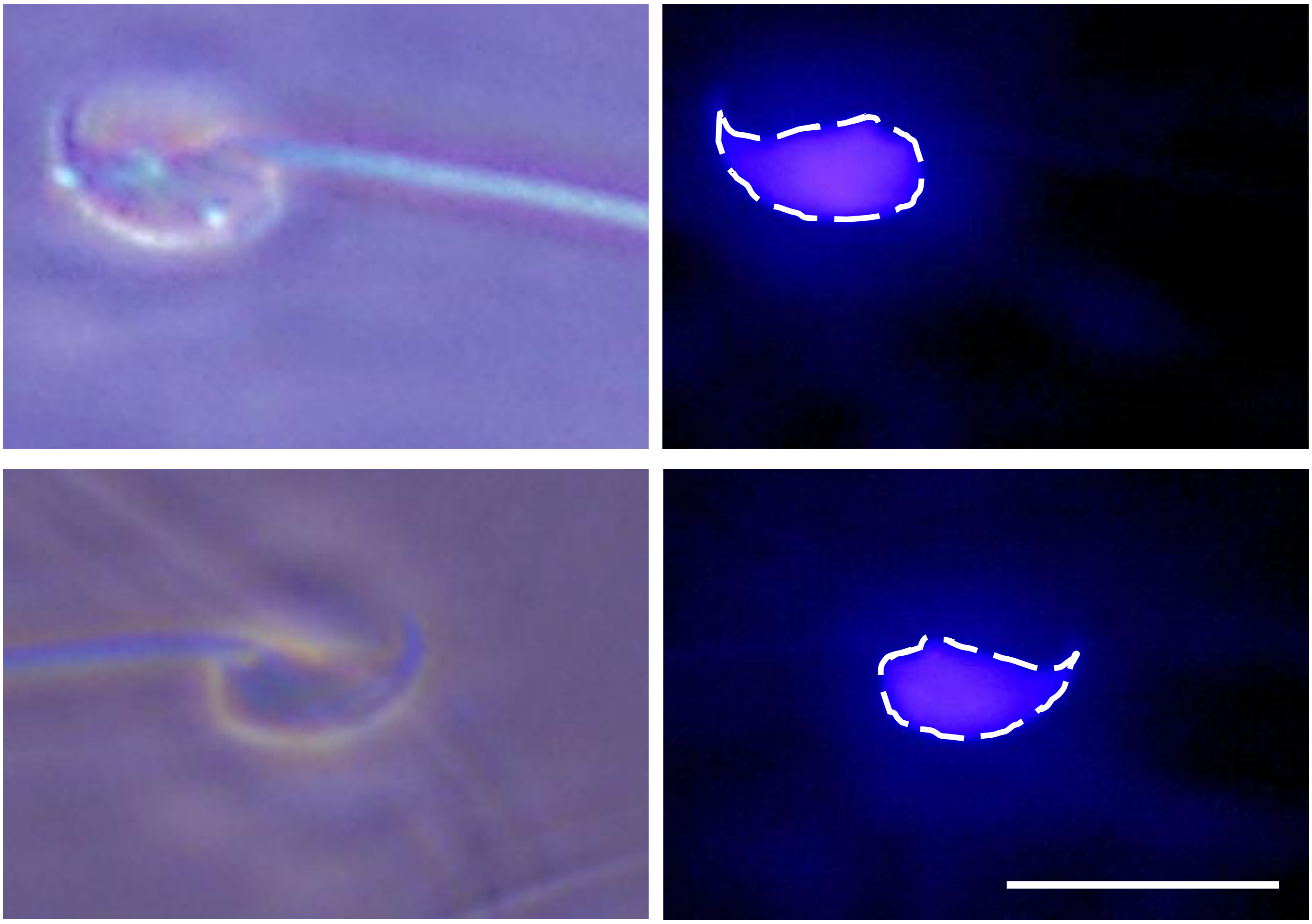

Bar: 10μm

S4 Figure

A

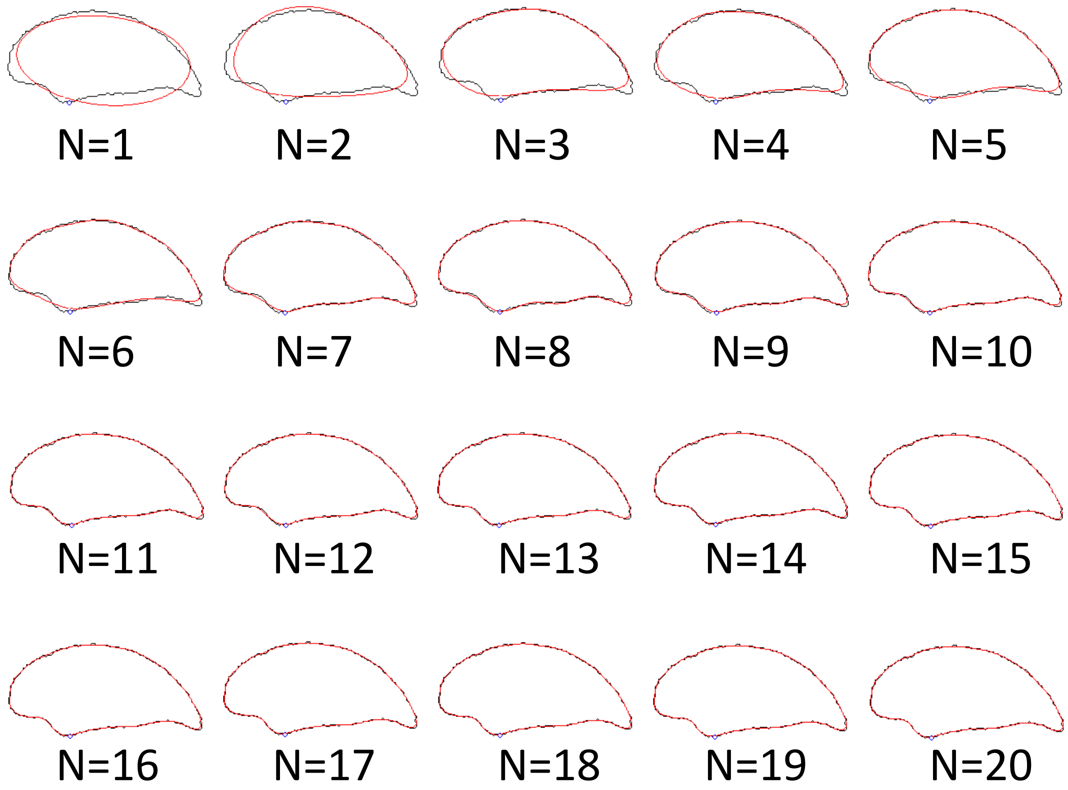
N=1
N=2
N=3
N=4
N=5
N=6
N=7
N=8
N=9
N=10
N=11
N=12
N=13
N=14
N=15
N=16
N=17
N=18
N=19
N=20

B

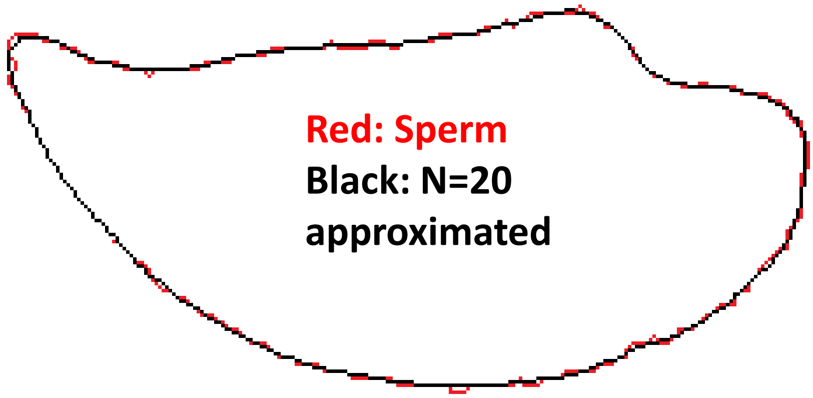
Red: Sperm
Black: N=20
approximated

S5 Figure

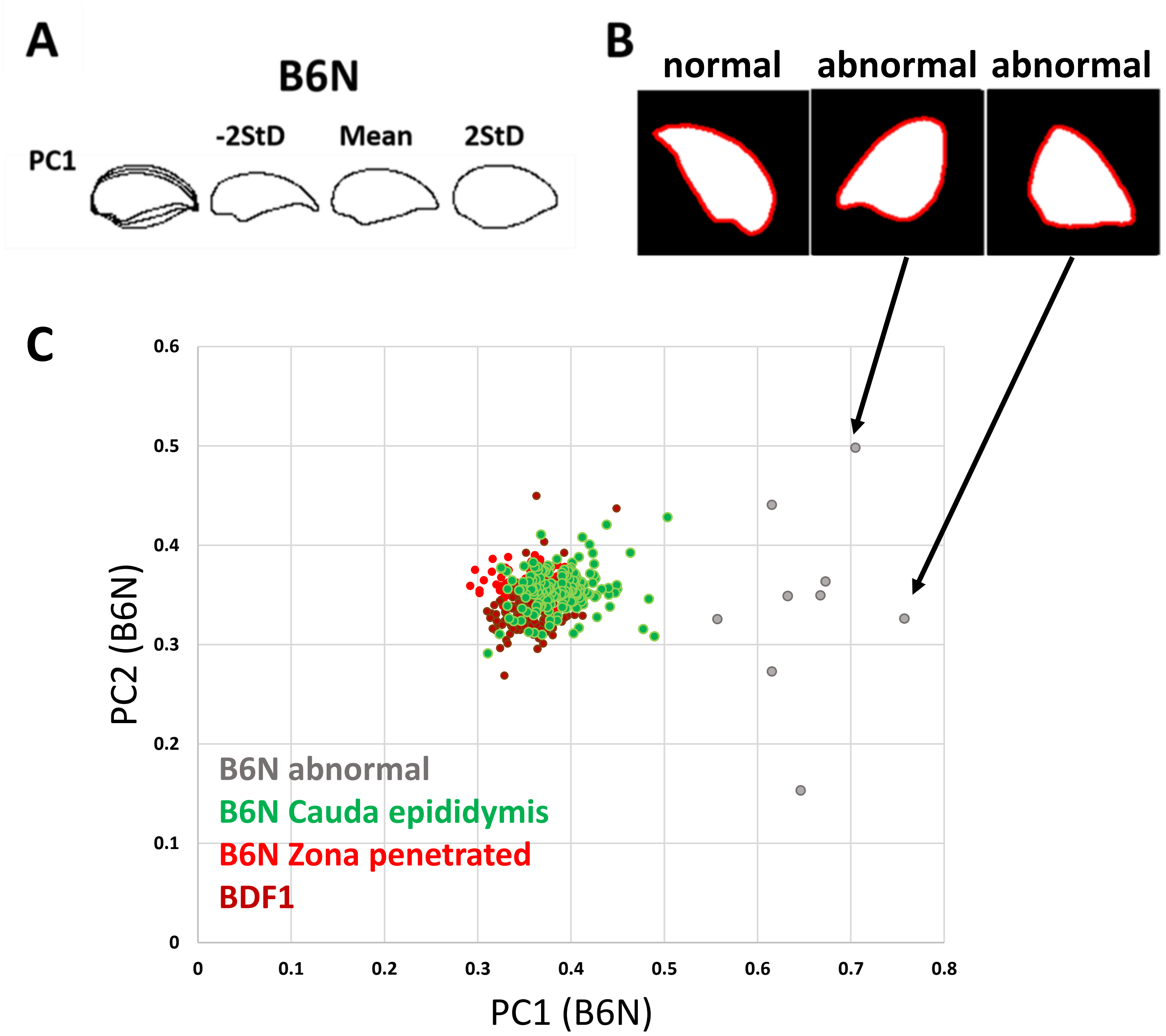
A
B6N
PC1
-2StD
Mean
2StD
B
normal
abnormal
abnormal
C
0.6
0.5
0.4
0.3
0.2
0.1
0
PC2 (B6N)
B6N abnormal
B6N Cauda epididymis
B6N Zona penetrated
BDF1
0
0.1
0.2
0.3
0.4
0.5
0.6
0.7
0.8
PC1 (B6N)

S6 Figure

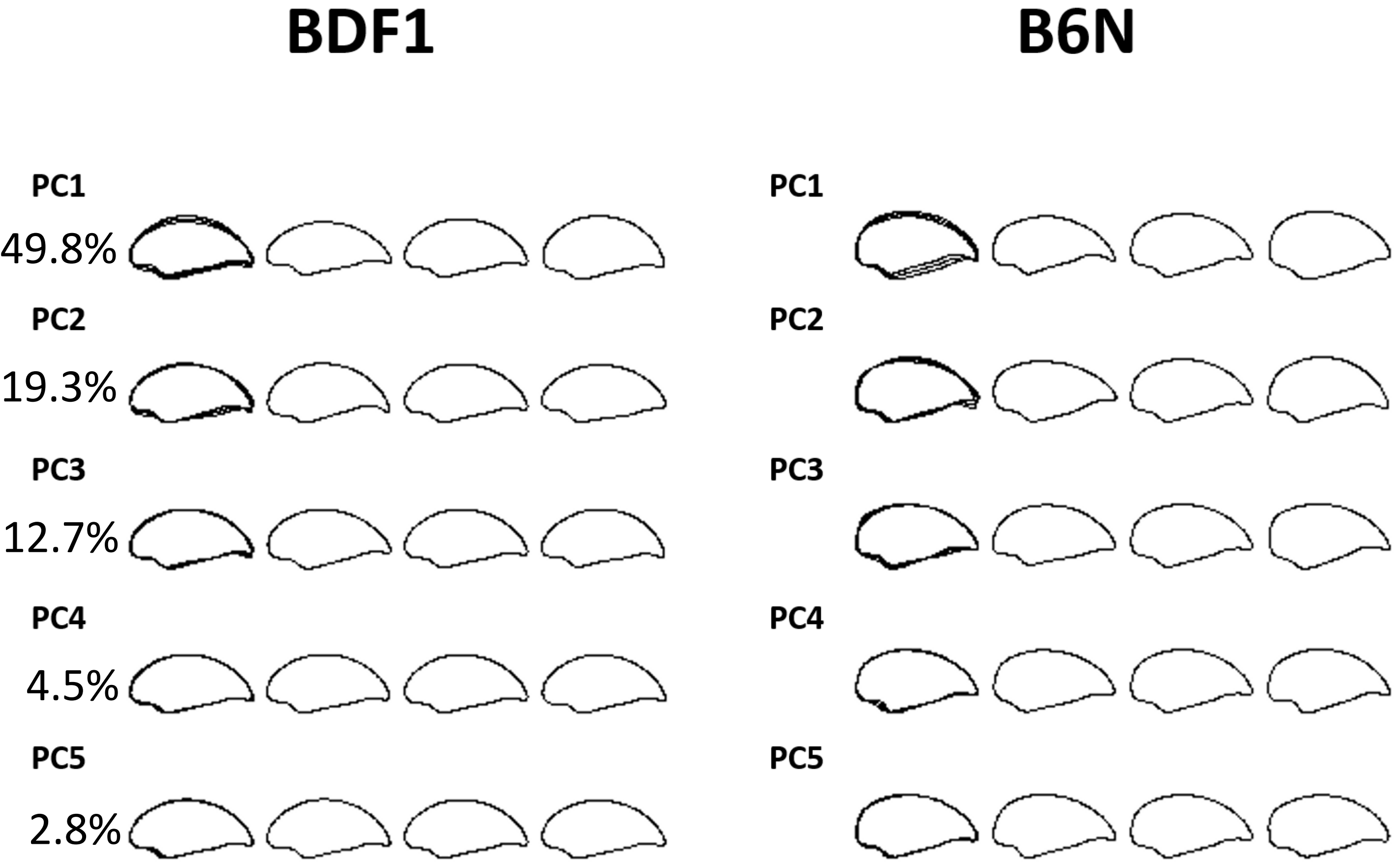
BDF1
B6N
PC1
49.8%
PC2
19.3%
PC3
12.7%
PC4
4.5%
PC5
2.8%
PC1
PC2
PC3
PC4
PC5

S7 Figure

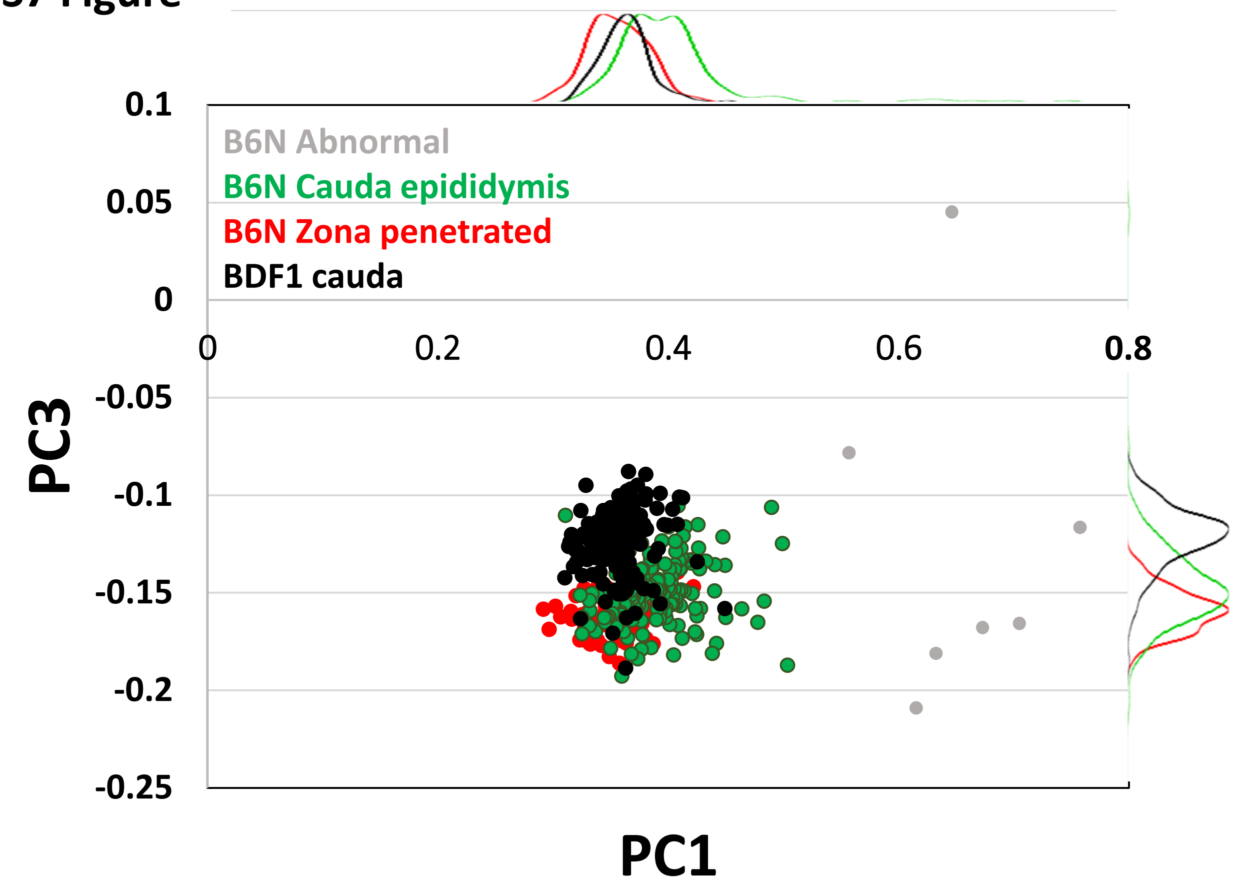

0.1
0.05
0
-0.05
-0.1
-0.15
-0.2
-0.25
0
0.2
0.4
0.6
0.8
B6N Abnormal
B6N Cauda epididymis
B6N Zona penetrated
BDF1 cauda
PC3
PC1

S8 Figure

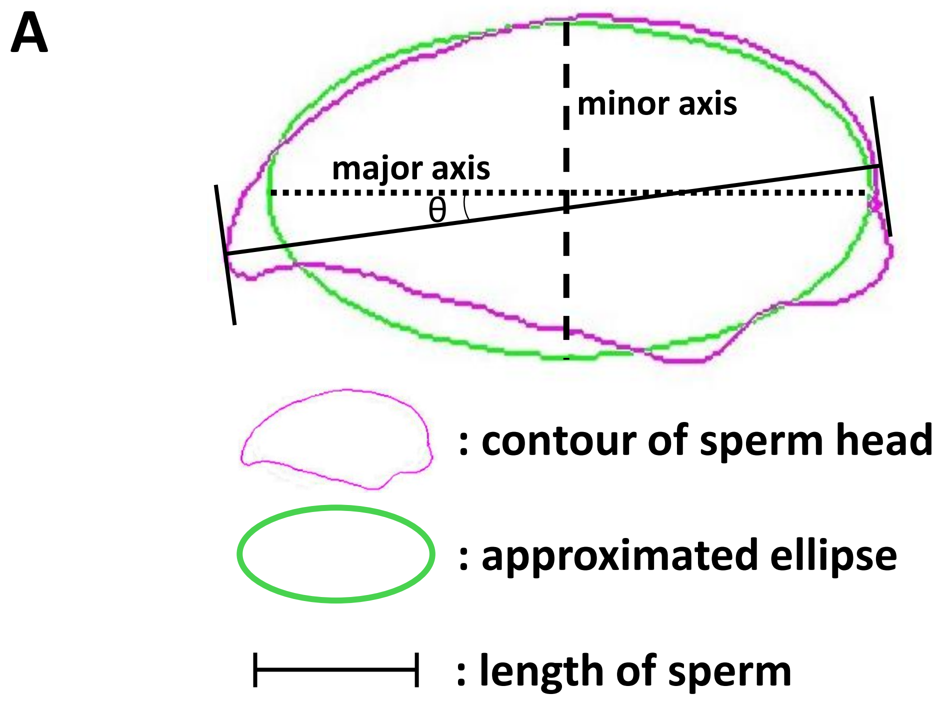
A
minor axis
major axis
θ
: contour of sperm head
: approximated ellipse
: length of sperm

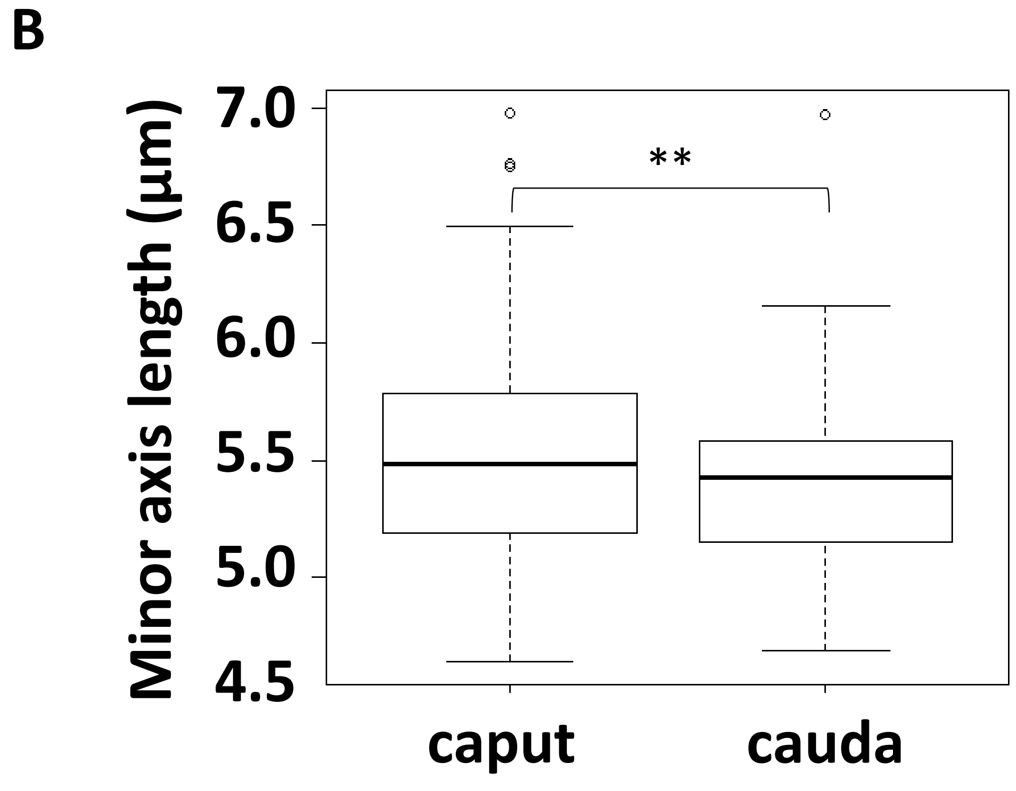
B
Minor axis length (μm)
7.0
6.5
6.0
5.5
5.0
4.5
**
caput
cauda

S9 Figure

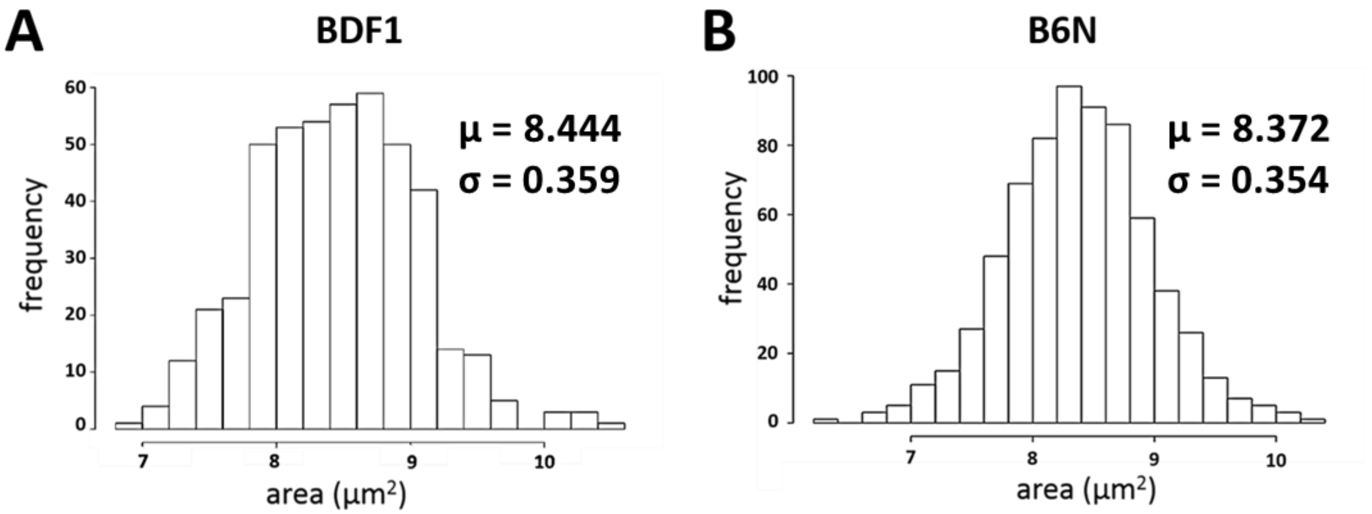
A
BDF1
μ = 8.444
σ = 0.359
frequency
0
10
20
30
40
50
60
7
8
9
10
area (μm2)
B
B6N
μ = 8.372
σ = 0.354
frequency
0
20
40
60
80
100
7
8
9
10
area (μm2)

S10 Figure

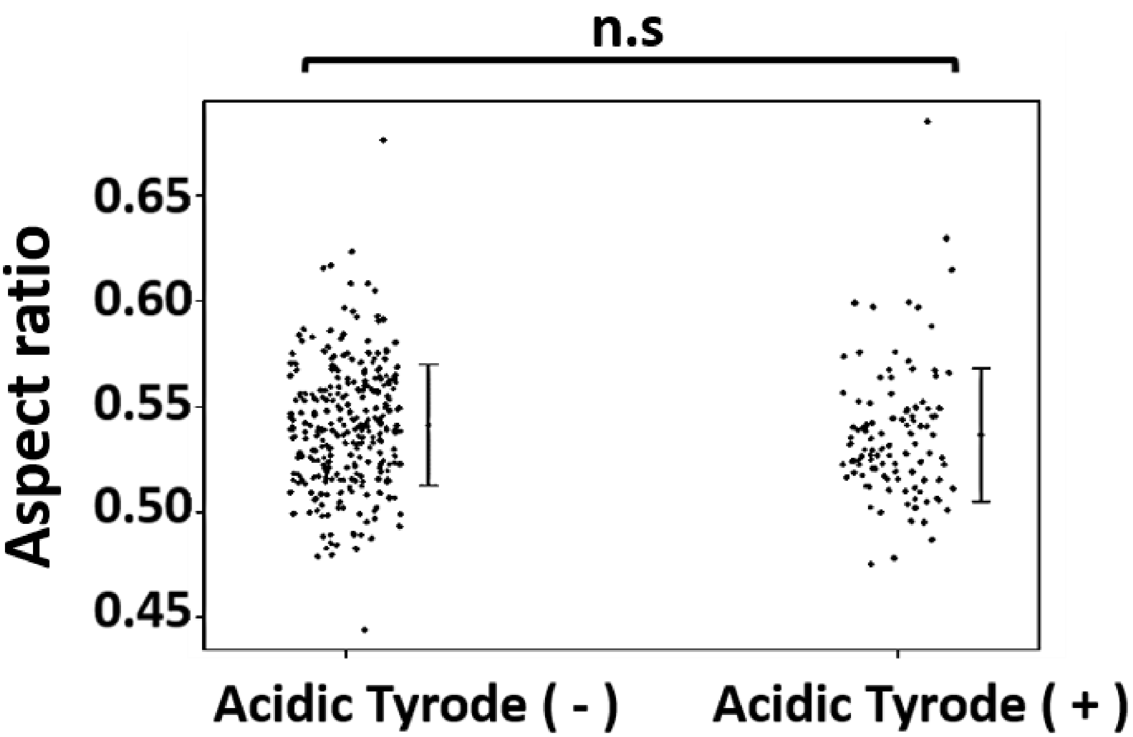
n.s
Aspect ratio
0.65
0.60
0.55
0.50
0.45
Acidic Tyrode (-)
Acidic Tyrode (+)
